# Host genetic variation in mucosal immunity pathways influences the upper airway microbiome

**DOI:** 10.1186/s40168-016-0227-5

**Published:** 2017-02-01

**Authors:** Catherine Igartua, Emily R. Davenport, Yoav Gilad, Dan L. Nicolae, Jayant Pinto, Carole Ober

**Affiliations:** 1 Department of Human Genetics, University of Chicago, Chicago, IL 60637 USA; 2 Department of Molecular Biology and Genetics, Cornell University, Ithaca, NY 14853 USA; 3 Department of Medicine, University of Chicago, Chicago, IL 60637 USA; 4 Department of Statistics, University of Chicago, Chicago, IL 60637 USA; 5 Section of Otolaryngology-Head and Neck Surgery, Department of Surgery, University of Chicago, Chicago, IL 60637 USA

**Keywords:** QTL mapping, Microbiome, Nasal, Upper airways, Host-microbe interactions, Gene-environment, GWAS

## Abstract

**Background:**

The degree to which host genetic variation can modulate microbial communities in humans remains an open question. Here, we performed a genetic mapping study of the microbiome in two accessible upper airway sites, the nasopharynx and the nasal vestibule, during two seasons in 144 adult members of a founder population of European decent.

**Results:**

We estimated the relative abundances (RAs) of genus level bacteria from 16S rRNA gene sequences and examined associations with 148,653 genetic variants (linkage disequilibrium [LD] *r* ^2^ < 0.5) selected from among all common variants discovered in genome sequences in this population. We identified 37 microbiome quantitative trait loci (mbQTLs) that showed evidence of association with the RAs of 22 genera (*q* < 0.05) and were enriched for genes in mucosal immunity pathways. The most significant association was between the RA of *Dermacoccus* (phylum Actinobacteria) and a variant 8 kb upstream of *TINCR* (rs117042385; *p* = 1.61 × 10^−8^; *q* = 0.002), a long non-coding RNA that binds to peptidoglycan recognition protein 3 (*PGLYRP3*) mRNA*,* a gene encoding a known antimicrobial protein. A second association was between a missense variant in *PGLYRP4* (rs3006458) and the RA of an unclassified genus of family Micrococcaceae (phylum Actinobacteria) (*p* = 5.10 × 10^−7^; *q =* 0.032).

**Conclusions:**

Our findings provide evidence of host genetic influences on upper airway microbial composition in humans and implicate mucosal immunity genes in this relationship.

**Electronic supplementary material:**

The online version of this article (doi:10.1186/s40168-016-0227-5) contains supplementary material, which is available to authorized users.

## Background

Diverse populations of microorganisms inhabit nearly every surface of the human body, and these complex assemblies of microbes reflect host-microbe and microbe-microbe interactions. Collectively, these microorganisms constitute the human microbiome [1]. Under healthy conditions, the relationship between microbes and the host is symbiotic with many physiologic benefits to the host [2]. Imbalances or changes in the composition of bacterial communities can shift this relationship from symbiotic to pathogenic, a condition known as dysbiosis, which has been implicated in a variety of diseases [3]. For example, altered composition of airway microbiota has been linked to important respiratory diseases such as sinusitis [4], chronic obstructive pulmonary disease (COPD) [5], and asthma [6–8]. Similar to the traits it influences, the microbiome itself can be considered a complex phenotype with environmental and genetic factors contributing to its composition [9]. Understanding how host genetic variation shapes the microbiome and how the microbiome ultimately functions to modulate host immunity are fundamental questions that are central to fully characterizing the architecture of many common diseases that occur at mucosal surfaces, including those involving the airway.

Although knowledge of the airway microbiome lags behind that of the gut, important characteristics of the microbial communities in the airway are beginning to emerge. Similar to the gut, the community structure of an individual’s airway microbiome is established early in life and plays a critical role in immune development [10–12]. Many external factors influence the airway microbiome, including mode of delivery at birth [13], breastfeeding [14], antibiotic use [15, 16], and exposure to tobacco smoke [17] and pathogens [18]. While the influences of environmental exposures on microbiome composition are well known, the degree to which host genetics plays a role in structuring microbial communities is less well understood. In fact, recent data suggest that host genetics may play an important role in shaping microbiome composition. For example, the heritability of the gut microbiome was recently investigated in 1,126 twin pairs [19]. Out of 945 taxa examined, the RAs of 8.8% of taxa had non-zero heritability estimates suggesting that the abundances of those bacteria are influenced by host genetic variation. Moreover, more similar microbiome structures among related individuals compared to unrelated individuals [20, 21] further support a role for genetics influencing interindividual variability in microbiome profiles. In fact, quantitative trait locus (QTL) approaches have successfully identified variation in candidate host genes that influence the RA of specific bacteria not only in *Drosophila* and mice but also in humans [22].

Studies of host genetic influences on the microbiome are particularly challenging due to the profound effects of environmental exposures on microbiome variability. It is not surprising, therefore, that two studies were unable to show host genotype effects on the human gut microbiome [23, 24]. Studies of related individuals and even twin pairs are confounded to a large extent by the more similar environments among close relatives, making it impossible to completely disentangle the relative roles of genes and environment. To address these challenges, we focused our studies on the Hutterites, a founder population that practices a communal, farming lifestyle that minimizes environmental variation between individuals [25], and should increase power to identify genetic influences on complex traits, including the airway microbiome composition. For example, Hutterites prepare and eat all meals in communal kitchens, smoking is prohibited and rare, and individual family homes are nearly identical within each colony (communal farm) and very similar across colonies. Furthermore, the Hutterites in our studies are related to each other in a 13-generation pedigree and are descendants of only 64 founders. Finally, nearly all genetic variation in these individuals has been revealed through whole genome sequencing studies in 98 Hutterite individuals [26].

We previously reported studies of the gut microbiome in the Hutterites [27, 28]. Here, we interrogated the interaction between host genetic variation and microbiome composition in two accessible sites in the upper airways, the nasal vestibule and the nasopharynx, which have important physiologic functions and relevance to airway diseases. While the nasal vestibule is located in the anterior nares and in direct contact with the environment, the nasopharynx is in the posterior nasal passage and continuous with the lower airway. Overall, our findings demonstrate that the airway microbiome is influenced by host genotype at many loci and suggest that host expression of innate and mucosal immune pathway genes plays a significant role in structuring the airway microbiome.

## Results

### Nasal microbiome composition

**Fig. 1:**
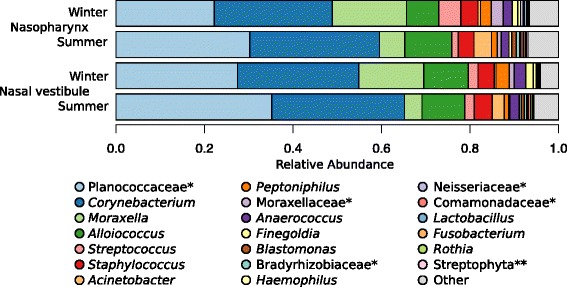
Taxonomic composition of bacterial communities in the nasal vestibule and the nasopharynx, sampled in summer and in winter. Genus level mean RA is shown for the 20 most abundant genera identified in the samples. The remaining 146 genera are grouped as “other”. Out of the six most abundant genera (83.30% of the sequences) in our sample, four (*Corynebacterium*, *Moraxella*, *Streptococcus*, and *Staphylococcus*) were among the most abundant (RA > 1%) in adult participants in the HMP anterior nares sample [1] (adults) and five (*Corynebacterium*, *Moraxella*, *Alloiococcus*, *Streptococcus*, and *Staphylococcus*) among infants in the Children Asthma Study nasopharynx sample [85].*Genus unclassified, family level presented. **Genus and family unclassified, order level presented

To characterize the variation of the microbiome from the nasal vestibule and the nasopharynx, we first analyzed 16S rRNA V4 gene sequences from 322 samples collected from 144 Hutterite adults in summer and/or in winter months (Table 1 and Additional file 1: Table S1). After applying quality control filters and subsampling to 250,000 reads per sample, 83 million reads were assigned to 563 operational taxonomic units (OTUs) with 97% sequence identity. We identified sequences from eleven phyla, with three accounting for 98.94% of the sequences—Firmicutes (52.28%), Actinobacteria (29.81%), and Proteobacteria (16.85%). We then classified OTUs into 166 genera; six dominant genera accounted for 83.30% of the sequences (Fig. 1 and Additional file 2: Table S1A).

**Table 1:** Sample composition: a total of 332 samples were collected from 144 (58 male, 86 female) Hutterite adults (age 16 to 78 years)

Nasal site	Summer	Winter	Both seasons	Unique subjects
Vestibule	87	80	34	133
Nasopharynx	88	77	40	125
Both sites	72	60	23	144

In a prior study in a largely overlapping sample of adult Hutterites, we identified large seasonal variation in the gut (fecal) microbiome [28]. To see if similar patterns were present in the nasal microbiome, we examined the genus level RAs for individuals studied in both seasons (*n* = 34 for the nasal vestibule and 40 for the nasopharynx). The RA of 12 genera in the nasal vestibule and 15 in the nasopharynx differed by season after applying a Bonferroni correction (paired Wilcox rank-sum test, *p* < 0.0003), nine of which were different between seasons at both nasal sites (Additional file 2: Table S1B, C). Similarly, we looked for genus level RAs that differed between the nasal sites within each of the two seasons (*n* = 72 for the summer and 60 for the winter) but did not identify statistically significant differences (Additional file 2: Table S1D, E).

### Nasal microbiome diversity

**Fig. 2:**
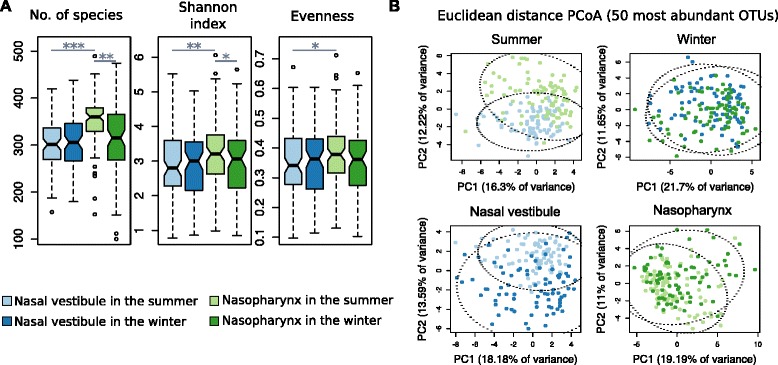
**a** Alpha diversity. Alpha diversity measurements for microbial communities from the nasal vestibule and the nasopharynx by season. The nasopharynx in the summer (*light green*) shows the overall largest alpha diversity. *Paired Wilcoxon rank-sum test *p* < 0.5; **p < 0.01; ***p < 0.001. **b** Beta diversity. Principal coordinate analysis (PCoA) of the 50 most abundant OTUs derived from Euclidean distance. Nasal site samples show separation in the summer (PC2 *p* < 2.2 × 10^−16^). Seasonal samples show separation in the nasal vestibule (PC2 *p* < 2.2 × 10^−16^) and in the nasopharynx (PC2 *p* < 0.0002). Ellipsoids contain 95% of the samples within each group

We used three diversity metrics to assess within sample (alpha) diversity for each of the four seasonal nasal site groups—the number of species (richness), Shannon index, and evenness. Overall, the highest alpha diversity was observed in the nasopharynx in the summer (Fig. 2a and Additional file 1: Figure S1A), where the number of observed species and the Shannon index reflected higher diversity compared to the nasopharynx in the winter (paired Wilcoxon signed-rank test; *p* = 0.002 and 0.048, respectively). Additionally, there was higher diversity in the nasopharynx in the summer compared to the nasal vestibule in the summer (paired Wilcoxon signed-rank test; richness *p* = 4.6 × 10^−7^, Shannon index *p* = 0.009, and evenness *p* = 0.031, respectively). Although higher diversity trends were observed in the nasopharynx in the summer compared to the winter, these associations were largely due to decreased alpha diversity among women compared to men in the winter (Wilcoxon signed-rank test; richness *p* = 0.03, Shannon index *p* = 0.002, and evenness *p* = 0.001, respectively; Additional file 1: Figure S1B). Lastly, Shannon index and evenness decreased with increasing age only in the nasopharynx in the summer (*p* = 0.019; Additional file 1: Figure S2).

We next analyzed community composition and structure between samples (beta diversity) by calculating Euclidean distances between all pairs of individuals. In the seasonal analyses, the summer samples for both the nasal vestibule and the nasopharynx had lower Euclidean distances compared to their respective winter samples (Wilcoxon signed-rank test, nasal vestibule *p* < 2.2 × 10^−16^, and nasopharynx *p* < 2.2 × 10^−16^), reflecting more similar microbiome diversity between pairs of individuals in the summer than in the winter. Moreover, Euclidean distances for the same individual paired with him/herself between seasons (separately within the nasal vestibule and nasopharynx samples) and between nasal sites (separately within the summer and the winter samples) were lower than the respective distances calculated between each individual with all other individuals (Wilcoxon signed-rank test nasal vestibule between seasons *p* = 9.25 × 10^−8^; nasopharynx between seasons *p* = 3.97 × 10^−12^; summer between nasal sites *p* < 2.2 × 10^−16^, winter between nasal sites *p* < 2.2 × 10^−16^; Additional file 1: Figure S3). These results reflect stability in microbiome structure between seasons and nasal sites within individuals, potentially reflecting a genetic component to microbiome composition and diversity.

To further examine the impact of anatomical location and season on beta diversity, we analyzed Euclidean distances derived from all OTUs with principal coordinate analysis (PCoA). Although PCoA did not show clear separation between the top two PCs and the four groups, analysis of similarity (ANOSIM) revealed significant differences in community structure (*p* < 9.9 × 10^−5^; *R* = 0.16). Because beta diversity was previously shown to have greater dissimilarities between anterior and others, more posterior nasal microbiomes [29], we performed PCoA again after selecting the 50 most abundant OTUs to determine if variation captured from composition of the most predominant bacteria would separate between nasal sites. In this analysis, PCoA showed clear dissimilarities between the NV and the NP in the summer (PC2 *p* < 2.2 × 10^−16^; ANOSIM *p* < 9.9 × 10^−5^; *R* = 0.24; Fig. 2b). We note that the lack of dissimilarity between nasal sites in the winter may result from decreased power due to the lower alpha diversity observed in the winter samples. In fact, the higher prevalence of respiratory viruses in temperate regions in this season [30] supports the possibility that viral-microbial interactions may synergistically affect microbial structure at both nasal sites in the winter. For example, studies of the airway microbiome after exposure to pH1NI [31] and rhinovirus [32] have demonstrated that viral infections can alter microbiome composition in a manner that decreases microbial diversity and enriches for pathogens associated with subsequent bacterial infections.

### Correlation between host genetic similarity and microbiome structure

**Fig. 3:**
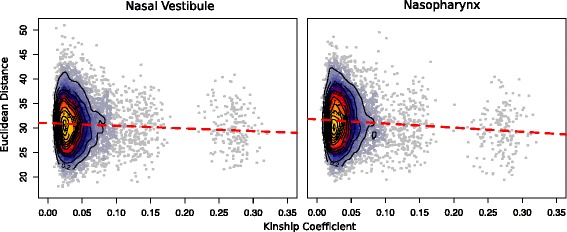
Heat scatterplots of Euclidean distance (beta diversity) by kinship coefficient. Individuals with larger kinship coefficients (more related) have more similar beta diversities (lower Euclidean distances). *Red dashed* represents the trend line from a linear model. Nasal vestibule *p* < 1 × 10^−4^; nasopharynx *p* = 4.0 × 10^−4^

To evaluate the relationship between genetic similarity (or relatedness) among pairs of individuals and the similarity of their nasal microbiomes, we compared genetic distance, measured by the kinship coefficient and beta diversity between all pairs of individuals in the sample combined across seasons (see methods). We reasoned that if there was a genetic influence on bacterial composition and diversity, more related individuals should have lower measures of beta diversity, reflecting more similar microbiomes. To assess significance, we performed 10,000 permutations for each of the two nasal sites. This analysis revealed a significant negative Spearman correlation between kinship and beta diversity (NV *p* < 1 × 10^−4^, NP *p* = 4.0 × 10^−4^; Fig. 3).

Although an individual’s microbiome composition is highly sensitive to the household environment [33], sharing of households by the first degree relatives did not significantly affect the correlation between beta diversity and kinship in our sample. To examine this directly, we removed all the first degree relatives who lived in the same household (three sibling pairs and their parents; 15 out of 175 first-degree relative pairs in the sample) and repeated the analysis. The correlation between kinship and Euclidean distance remained significant (NV *p* < 1 × 10^−4^ and NP *p* = 5.0 × 10^−4^), indicating that the significant effect of kinship on microbiome similarities between Hutterite adults is not likely due to shared environments. Instead, we attribute these correlations largely to shared genetic variation.

### Genome-wide association studies of relative abundance

To directly test for host genetic effects on genus level bacteria in the nasal vestibule and in the nasopharynx, we performed microbiome quantitative trait locus mapping on the bacterial RAs for each nasal site in the summer sample and winter samples separately, and in a larger sample combining both seasons. We tested for associations between 52 and 90 genera with 148,653 SNPs (LD *r* ^2^ < 0.5) using a linear mixed model as implemented in GEMMA [34] and included sex and age as fixed effects and kinship as a random effect to adjust for the relatedness between all pairs of individuals in our study. Due to the multiple testing burden inherent in microbiome studies, genome-wide significance thresholds were determined within each bacteria GWAS. We note, however, that none of the associations passed a study-wide significance threshold (*p* < 1.1 × 10^−10^) that would correct for the number of bacteria, sites, and seasons tested. Our analyses revealed 37 mbQTLs at *q* < 0.05, eight of which passed genome-wide significance (*p* < 5 × 10^−8^), and three of which were associated with multiple bacteria (overall 37 variants associated with 22 genus level bacteria). Of the 37 mbQTLs, 14 were associated with 10 genera in the nasal vestibule and 23 were associated with 14 genera in the nasopharynx. The results for mbQTLs with *q* < 0.05 are shown in Table 2, and results for 108 mbQTLs with *q* < 0.10 are shown in Additional file 3: Table S2. In addition to mapping the relative abundance, we also mapped alpha and beta diversity, the results of which are shown in Additional files 4 and 5: Tables S3 and S4.

**Table 2:** QTL mapping results of nasal microbiome relative abundance

A. Nasal vestibule									
Bacteria genus (phylum/class/order/family)	Mean RA	rsID	Chr	Start	Alleles	Gene(s)	*p*	*q*	Beta
Summer									
*Dermacoccus* (Actinobacteria/Actinobacteria/Actinomycetales/Dermacoccaceae)	7.9 × 10^−5^	rs67386870	5	126156219	A/C	*LMNB1*	2.46 × 10^−7^	0.016	−1.10
		rs77536542	5	168583325	G/A	*SLIT3* (1,2)	6.35 × 10^−8^	0.005	−1.27
		rs117042385^a^	19	5530692	T/C	*ZNRF4*, *TINCR*	1.61 × 10^−8^	0.002	−1.16
		rs28362459	19	5844792	A/C	*FUT3*	9.47 × 10^−7^	0.047	−0.81
Unclassified genus (Actinobacteria/Actinobacteria/Actinomycetales/Micrococcaceae)	4.1 × 10^−4^	rs111354832	4	7136504	–/CAT	*FLJ36777*, *SORCS2*	5.99 × 10^−8^	0.015	−0.74
Winter									
*Kocuria* (Actinobacteria/Actinobacteria/Actinomycetales/Micrococcaceae)	5.2 × 10^−4^	rs12713689^a^	2	70427457	G/A	*C2orf42*, *TIA1*	2.10 × 10^−8^	0.005	−0.91
*Aerococcus* (Firmicutes/Bacilli/Lactobacillales/Aerococcaceae)	2.2 × 10^−4^	rs10505338	8	119755490	A/G	*SAMD12-AS1*, *TNFRSF11B*	2.02 × 10^−7^	0.038	−0.81
*Lactobacillus* (Firmicutes/Bacilli/Lactobacillales/Lactobacillaceae)	1.4 × 10^−3^	rs4142162	13	81127842	G/A	*SPRY2*, *LINC00377*	1.45 × 10^−7^	0.017	−0.77
Combined									
Unclassified genus (Actinobacteria/Actinobacteria/Actinomycetales/Intrasporangiaceae)	9.2 × 10^−5^	rs11085969	19	15792546	A/G	*CYP4F12*	5.84 × 10^−8^	0.005	−0.91
*Microbispora* (Actinobacteria/Actinobacteria/Actinomycetales/Micrococcaceae)	5.5 × 10^−4^	rs2891405	12	113152097	G/A	*MIR1302-1*, *RPH3A*	1.50 × 10^−7^	0.038	−0.81
Unclassified genus (Actinobacteria/Actinobacteria/Actinomycetales/Micrococcaceae)	3.5 × 10^−4^	rs111354832^a^	4	7136504	–/CAT	*FLJ36777*, *SORCS2* (2)	3.45 × 10^−8^	0.017	−0.77
*Peptoniphilus* (Firmicutes/Clostridia/Clostridiales/Tissierellaceae)	8.9 × 10^−5^	rs9865782	3	113652774	A/G	*GRAMD1C*	1.17 × 10^−7^	0.005	−0.91
*Paracoccus* (Proteobacteria/Alphaproteobacteria/Rhodobacterales/Rhodobacteraceae)	6.5 × 10^−5^	rs9953410	18	29532946	C/A	*TRAPPC8*, *RNF125*	2.21 × 10^−7^	0.038	−0.81
*Enterobacter* (Proteobacteria/Gammaproteobacteria/Enterobacteriales/Enterobacteriaceae)	6.4 × 10^−5^	rs11042877	11	10576232	A/C	*MRVI1-AS1*	3.86 × 10^−7^	0.017	−0.77
		rs12446497	16	7341674	A/G	*RBFOX1*	5.84 × 10^−7^	0.005	−0.91
B. Nasopharynx									
Bacteria species (phylum/class/order/family)	Mean RA	rsID	Chr	Start	Alleles	Gene(s)	*p*	*q*	Beta
Summer									
*Aerococcus* (Firmicutes/Bacilli/Lactobacillales/Aerococcaceae)	6.5 × 10^−4^	rs7702475^a^	5	58088523	A/G	*RAB3C*	3.97 × 10^−8^	0.010	0.77
Unclassified genus (Firmicutes/Bacilli/Lactobacillales/Aerococcaceae)	5.3 × 10^−4^	rs11888528	2	120118764	C/T	*C2orf76*	1.43 × 10^−7^	0.038	0.79
*Methylobacterium* (Proteobacteria/Alphaproteobacteria/Rhizobiales/Methylobacteriaceae)	4.3 × 10^−4^	rs308961	3	12150014	T/G	*SYN2*	8.12 × 10^−7^	0.034	−0.70
		rs10547084	4	37753111	–/TCTC	*RELL1*,*PGM2*	4.93 × 10^−7^	0.031	0.77
		rs67737950	4	40260058	G/C	*RHOH*, *LOC101060498*	2.73 × 10^−7^	0.023	0.85
		rs7702475	5	58088523	A/G	*RAB3C*	2.46 × 10^−7^	0.023	0.77
		rs1278260	10	127731197	C/A	*ADAM12*	6.21 × 10^−7^	0.031	0.95
Unclassified genus (Proteobacteria/Alphaproteobacteria/Caulobacterales/Caulobacteraceae)	8.5 × 10^−4^	rs927984	6	25412987	T/C	*LRRC16A*	2.78 × 10^−7^	0.036	0.94
		rs1543603^a^	6	25413922	A/G	*LRRC16A*	2.30 × 10^−8^	0.006	0.88
Winter									
*Mycobacterium* (Actinobacteria/Actinobacteria/Actinomycetales/Mycobacteriaceae)	2.2 × 10^−4^	rs1802665	10	61788623	G/T	*ANK3*	8.73 × 10^−8^	0.007	−0.98
*Gemella* (Firmicutes/Bacilli/Gemellales/Gemellaceae)	3.7 × 10^−4^	rs17631306^a^	1	111072322	A/G	*KCNA10*, *KCNA2*	2.94 × 10^−8^	0.008	1.27
Unclassified genus (Firmicutes/Bacilli/Lactobacillales)	5.7 × 10^−4^	rs1153741	2	182860422	G/A	*PPP1R1C*	1.52 × 10^−7^	0.039	−0.83
Combined									
*Gordonia* (Actinobacteria/Actinobacteria/Actinomycetales/Gordoniaceae)	1.1 × 10^−4^	rs61925863	12	66694722	C/G	*IRAK3*, *HELB*	2.88 × 10^−7^	0.038	−0.90
		rs12435212^a^	14	85483485	G/T	*LINC00911*	1.93 × 10^−8^	0.005	0.88
Unclassified genus (Firmicutes/Clostridia/Clostridiales/Clostridiaceae)	1.0 × 10^−2^	rs9661504	1	205915667	A/T	*SLC26A9*, *FAM72C*	4.16 × 10^−7^	0.046	−0.70
		rs10232599	7	46035291	G/A	*IGFBP3*, *TNS3*	1.14 × 10^−7^	0.029	−0.67
Unclassified genus (Actinobacteria/Actinobacteria/Actinomycetales)	1.3 × 10^−3^	rs12156316	8	41706484	T/C	*ANK1*	8.16 × 10^−7^	0.042	0.58
		rs10901086	9	134635034	T/C	*RAPGEF1*, *MED27*	7.05 × 10^−7^	0.042	0.89
		rs12244238	10	6083239	G/A	*IL2RA*	6.82 × 10^−7^	0.042	−0.69
Unclassified genus (Actinobacteria/Actinobacteria/Actinomycetales/Micrococcaceae)	5.0 × 10^−4^	rs3006458	1	153320372	T/G	*PGLYRP4*	5.10 × 10^−7^	0.032	−0.75
		rs4774283	15	58114121	T/G	*GCOM1*, *ALDH1A2*	2.56 × 10^−7^	0.032	−0.57
		rs4814474	20	16322199	A/C	*KIF16B*	9.3 5× 10^−7^	0.040	0.56
*Rhodococcus* (Actinobacteria/Actinobacteria/Actinomycetales/Nocardiaceae)	6.4 × 10^−5^	rs1653301^a^	2	201076401	A/G	*C2orf47, SPATS2L*	1.45 × 10^−8^	0.004	−0.82
Unclassified genus (Actinobacteria/Actinobacteria/Actinomycetales/Sporichthyaceae)	1.5 × 10^−4^	rs13128830	4	21455808	T/C	*KCNIP4*	3.03 × 10^−7^	0.022	0.64
Unclassified genus (Proteobacteria/Alphaproteobacteria/Sphingomonadales/Sphingomonadaceae)	5.1 × 10^−3^	rs1653301	2	201076401	A/G	*C2orf47, SPATS2L* (3)	9.48 × 10^−8^	0.024	−0.80

A. Nasal vestibule. Fourteen host variants were associated at a *q* < 0.05 with the relative abundance of 10 genera. rs111354832 is associated with an unclassified genus of family Micrococcaceae in the summer and in the combined sample. B. Nasopharynx. Twenty-three host variants were associated at a *q* < 0.05 with the relative abundance of 14 genera. At this site, 2 SNPs (rs1653301 and rs7702475) are associated with more than one bacterium. rsIDs presented for dbSNP142. ^a^Genome-wide significant result (*p* < 5 × 10^−8^). Alleles presented as minor/major. Direction of effect is presented for the minor allele. *RA* relative abundance, *Chr* chromosome. Gene labels 1–4 correspond to genes previously reported in either the Bonder [55] or Goodrich [19] GWAS. 1, Goodrich (fecal; unclassified genus of family Clostridiaceae); 2, Bonder (fecal; PWY-6948_sitosterol_degradation_to_androstenedione); 3, Goodrich (fecal; genus *Blautia*)

**Fig. 4:**
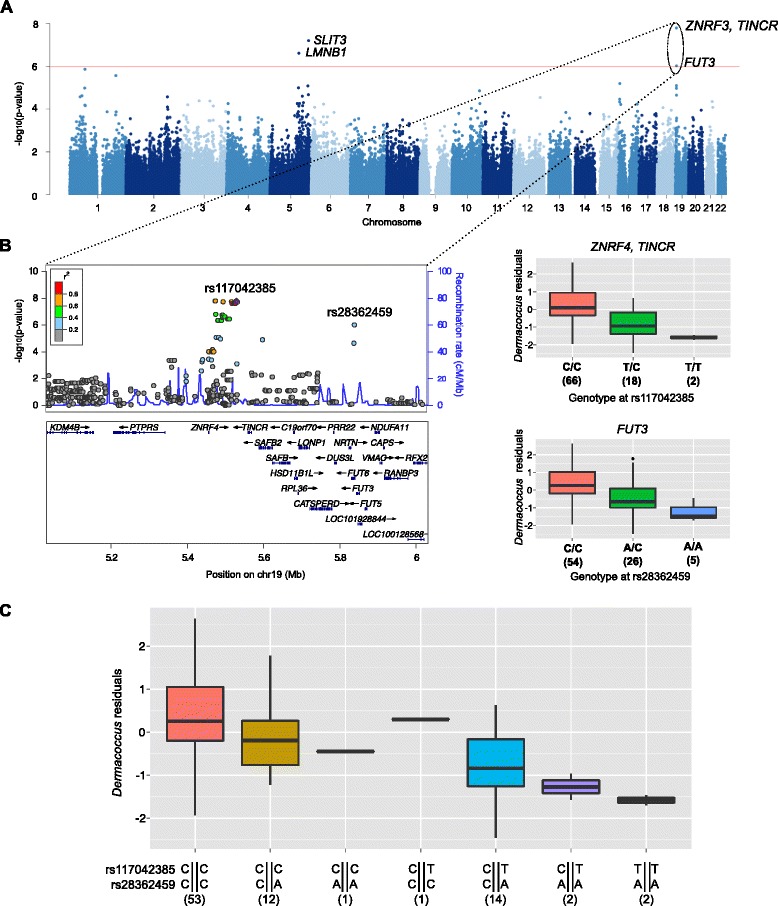
Associations with the RA of *Dermacoccus* in the nasal vestibule in the summer. **a** Manhattan plot. Association results are presented for variants pruned for LD (*r* ^2^ < 0.5). Four variants on chromosomes 5 and 19 are associated with the RA of *Dermacoccus* at a *q* < 0.05 significance threshold (*red line*). **b** Locus and genotype plots for the 2 mbQTLs on chromosome 19. Variants included in the locus plot are those with MAF > 10% in the Hutterites, prior to LD pruning. Genotype plots show both minor alleles (T at rs117042385 and A at rs28362459) are associated with lower *Dermacoccus* RA. **c** *Box plots* of *Dermacoccus* residuals for rs117042385 and rs28362459 phased haplotypes. *Numbers underneath each boxplot* represent the number of individuals in each genotype or haplotype class

The most significant association with relative abundance was with an intergenic SNP 8 kb upstream of the *TINCR* gene on chromosome 19 and the abundance of *Dermacoccus* (phylum Actinobacteria) in the nasal vestibule in the summer (rs117042385; *p* = 1.61 × 10^−8^; *q* = 0.002, Fig. 4a, b). *Dermacoccus* abundance has been observed to be depleted in the skin of individuals with atopic dermatitis [35], and microbes belonging to the Actinobacteria phylum are characterized by their production of metabolites with anti-inflammatory and antimicrobial properties [36]. Interestingly, *TINCR* is a long non-coding RNA gene that controls human epidermal differentiation and directly binds to the peptidoglycan recognition protein 3 (*PGLYRP3*) transcript [37]. These combined results reflect the important role of *Dermacoccus* in surface community structure and barrier integrity in both the skin and nasal epithelia [38]. A second mbQTL (rs28362459), located 314 kb downstream from the *TINCR* mbQTL (*r* ^2^ = 0.26; *D* = 0.76), was also associated with the RA of *Dermacoccus* in the same site and season as *TINCR* (*p* = 9.47 × 10^−7^, *q =* 0.047, Fig. 4a, b). rs28362459 is a missense variant in fucosyltransferase 3 (*FUT3*), a gene essential for the synthesis of Lewis blood groups [39, 40].

To determine if the association of increased *Dermacoccus* RA with the rs28362459-C allele in *FUT3* is independent of the association with the rs117042385-C allele upstream of *TINCR*, we phased the two variants and examined the four haplotypes (seven diplotypes) present in our sample*.* This revealed independent effects of genotypes at both SNPs contributing to the RA of *Dermacoccus* (*p* = 4.65 × 10^−9^; Fig. 4c). In particular, individuals who were homozygous for both alleles (rs117042385-CC/rs28362459-CC) had the highest RA of *Dermacoccus*, while one or two copies of the *FUT3* rs28362459-A allele on a homozygous *TINCR* rs117042385-CC background was associated with decreased RA. Overall, the presence of a *FUT3* rs28362459-A allele was associated with lower RA regardless of genotype at *TINCR* rs117042385. The two individuals who were homozygous for both the *TINCR* rs117042385-T and *FUT3* rs28362459-A alleles did not have any *Dermacoccus* sequences detected. Overall, these results suggest that the *FUT3* rs28362459 and *TINCR* rs117042385 variants (or variants in strong LD with them) are exerting independent effects on the RA of *Dermacoccus*.

Another mbQTL that linked PGLYRP genes more directly to host regulation of the microbiome is an association between a missense variant in *PGLYRP4* (rs3006458) on chromosome 1 and the RA of an unclassified genus of family Micrococcaceae (phylum Actinobacteria) in the combined season nasopharynx sample (*p* = 5.10 × 10^−7^; *q =* 0.032, Additional file 1: Figure S4A). This same SNP was also associated with genus *Aerococcus* in the nasopharynx in the winter at a less stringent *q* value cutoff (phylum Firmicutes; *p* = 1.28 × 10^−6^; *q* = 0.06). Peptidoglycan recognition proteins (PGRPs) are a conserved family of antibacterial pattern recognition molecules that directly bind peptidoglycan and other bacterial cell wall components, including lipopolysaccharide (LPS) [41]*.* The genus *Aerococcus* is commonly found in a wide range of environments including air, dust, soil, meat-cutting brines, marine sources, and the human respiratory tract [42]. Other lactic acid bacteria belonging to the order Lactobacillales (e.g., *Streptococcus pneumoniae*) colonize mucosal surfaces in the upper and lower airways of the host and cause important respiratory diseases including sinusitis, acute otitis media, and pneumonia [43].

**Fig. 5:**
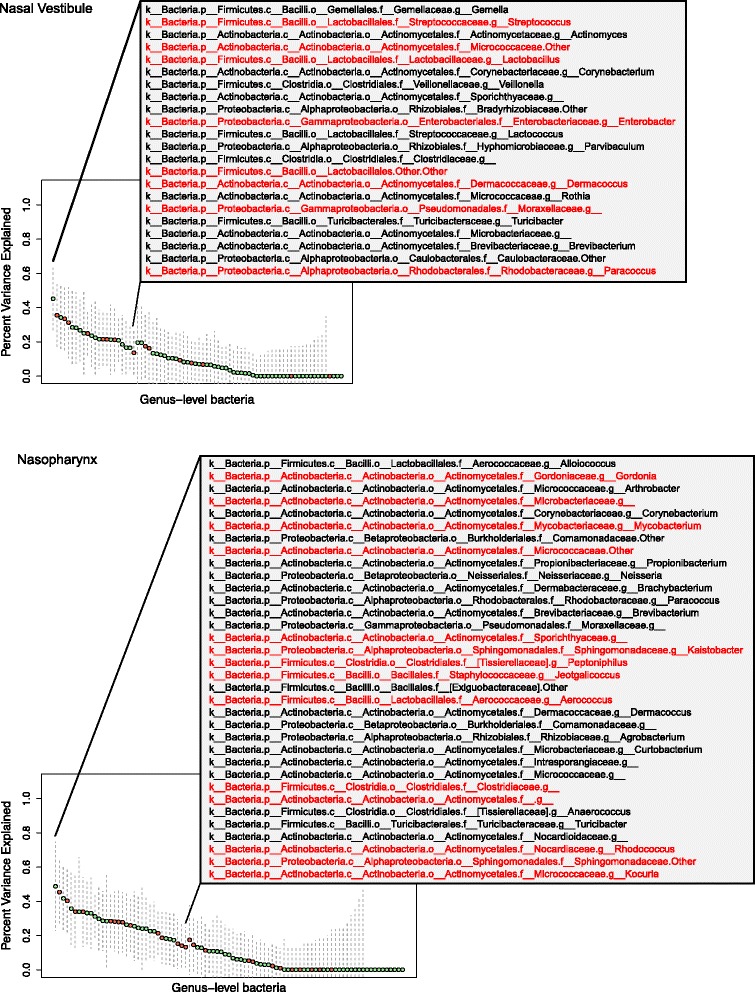
SNP heritability estimates for 90 bacteria tested in the nasal vestibule and nasopharynx combined samples. *Each point* represents the percent variance explained (PVE) for each bacteria genus level relative abundance; *dashed lines* show the standard error. The bacteria listed in the *inset* have SNP heritability estimates with standard errors that do not span zero. Bacteria for which mbQTLs (*q* < 0.10) were identified in either the seasonal or the combined samples are shown in *red*. Heritability estimates for all bacteria are presented in Additional file 4: Table S4

For each of the 90 bacteria tested, we calculated the proportion of relative abundance variance explained (PVE) by the joint effect of all SNPs tested in the mapping analyses (SNP heritability). PVE estimates the maximum phenotypic variance that can be explained by the variants interrogated in the analysis and provides a lower bound heritability estimate. In the combined analyses, there were 22 bacteria in the nasal vestibule (27%) and 34 bacteria in the nasopharynx (37%) that had SNP heritability estimates with standard errors not intersecting with zero. These estimates ranged between 13.25 and 48.69% (Fig. 5 and Additional file 6: Table S5). Overall, bacteria with identified mbQTLs in this study (*q* < 0.10) had higher estimates of SNP heritability than the remaining tested bacteria (Wilcoxon signed-rank test NV *p* = 0.009; NP *p* = 0.05).

### m*bQTL* associations *with multiple bacteria*

Five mbQTLs (*q* < 0.05), including the *PGLYRP4* mbQTL discussed above, had associations with multiple bacteria within the same nasal site at a relaxed significance threshold (*q* < 0.10). The largest number of associations identified with a single mbQTL was an intronic variant in the leucine rich repeat containing 16A (*LRRC16A*; rs1543603) and the RAs of five Proteobacteria in the nasopharynx in the summer (unclassified genus of family Caulobacteracea, unclassified genus of family Bradyrhizobiaceae, *Parvibaculum*, *Blastomonas*, and *Rheinheimera*; average cumulative sum = 2.1% of all RA and 13.5% of all Proteobacteria), of which Caulobacteraceae was the most significant (*p* = 2.30 × 10^−8^, *q* = 0.006; Additional file 1: Figure S4B). *LRRC16A* encodes CARMIL (capping protein, Arp2/3, and Myosin-I linker), a protein that plays an important role in cell shape and motility [44].

**Fig. 6:**
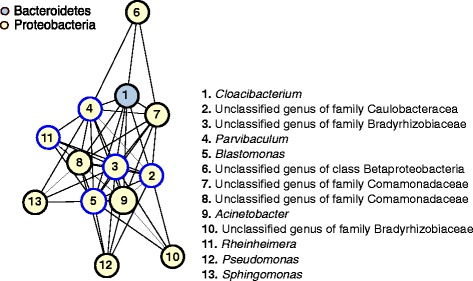
Five genus level bacteria associated with rs1543603 (in *LRRC16A*) are hubs in a co-occurrence module of 12 Proteobacteria and one Bacteroidetes. Co-occurrence networks built from correlation coefficients between all 90 genus level RAs determined in the nasopharynx summer sample. *Nodes* represent bacteria and are listed by number, colored by phylum, and sized proportionally to the RA of each bacterium. *Edges* represent correlations greater than 0.75. *Blue node borders* represent the five bacteria associated with rs1543603, an intronic variant in *LRRC16A*

The association between genotype at a single SNP, rs1543603, with the RAs of five genus level bacteria suggested potential functional community level relationships between these five Proteobacteria. Indeed, the RAs of all five Proteobacteria were correlated with each other (correlation coefficients > 0.773; median 0.924). Four of the Proteobacteria were classified as Alphaproteobacteria and one as Gammaprotebacteria, classes of bacteria with pathogenic species (e.g., *Inquilinus*, and *Moraxella* and *Haemophilus*, respectively) that have been linked to clinically important airway diseases such as cystic fibrosis [45], COPD [46, 47], sinusitis [48], or asthma [7]. A co-occurrence network [49] assigned all five bacteria to a single network that included 13 bacteria (12 Proteobacteria [two Alphaproteobacteria, two Gammaproteobacteria, and three Betaproteobacteria] and one Bacteroidetes) from among the 90 genera tested in the nasopharynx in the summer (Fig. 6). In this network, genera from families Bradyrhizobiaceae and Caulobacteraceae*,* two of the five bacteria associated with rs1543603 (*p* = 3.25 × 10^−7^ and 2.30 × 10^−8^, respectively) are the largest hubs with nine neighbors each. We next tested for the association of the sum of these 13 correlated bacteria with rs1543603, but this metric was not more significant (*p* = 7.72 × 10^−5^) than any of the individual bacteria associations. These findings suggest that host genetic effects can act to modulate microbial community patterns, by directly affecting host-microbe interactions with only one or a few main drivers of the community.

### Pathway analyses of genes near mbQTLs

**Fig. 7:**
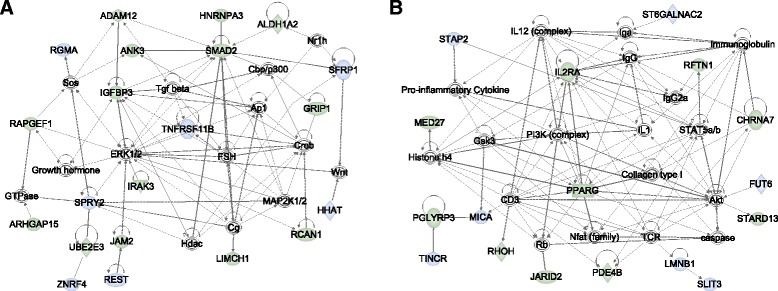
Ingenuity Pathway Analysis (IPA) interaction networks. Networks show genes near nasal mbQTLs are enriched for mucosal immunity pathways. Two significant networks (*p* < 10^−25^) are presented. **a** Network one is centered on SMAD2 and ERK1/2 (*p* < 10^−43^; 21 genes). **b** Network two is a highly connected network centered on IL2RA, STAT5a/b, and IL12, among others. This network contains many of the key regulators of mucosal immunity (*p* < 10^−29^; 17 genes). *Node color* represents genes near microbiome QTL associations in the nasal vestibule (*blue*) or in the nasopharynx (*green*); *open symbols* are genes added by IPA. *Edges* represent direct (*solid*) and indirect (*dashed*) interactions in the IPA Knowledge Base database. *Node shapes* correspond to functional classes of gene products: *concentric circles* for groups or complexes, *diamonds* for enzymes, *rectangles* for transcriptional regulators or modulators, *ovals* for trans-membrane receptors, and *circles* for other

To further understand how host genetic variation regulates nasal microbiome composition and to identify shared pathways among the mbQTLs identified in this study, we selected the closest gene to each mbQTL (*q* < 0.10; 131 genes) and to all variants in LD (*r* ^2^ > 0.8) with each mbQTL, using LD estimates in the Hutterites. We then generated protein-protein interaction networks among these genes, using Ingenuity Pathway Analysis Knowledge Base (IPA®, QIAGEN Redwood City, CA), a curated database of biological interactions and functional annotations. IPA identified two networks with Fisher’s exact *p* < 10^−25^. The most significant network included 21 of the 131 genes, nine of which were near mbQTLs with *q* < 0.05 (Fisher’s exact *p* = 10^−43^; Fig. 7a). This network contained many hubs including *SMAD2*, a gene that regulates the production of immunoglobulin A (IgA) by LPS-activated B-cells and activates immune response at other mucosal surfaces upon stimulation by pathogenic microbes [50]*.* The second significant network (Fisher’s exact *p* = 10^−29^; Fig. 7b) contained 17 of the 131 genes, also with nine genes near mbQTLs with *q* < 0.05. Many of the hubs in this network represent important modulators of mucosal immunity, including immunoglobulins A and G (IgG and IgG2a), IL12/IL12RA, TCR, and STAT5A/B [51–53].

### Comparison of genes near mbQTLs in the Hutterites to published microbiome QTL studies

To determine if genes associated with the RA of bacteria in the upper airway also influence the RA of bacteria at other human body sites, we compared 53 genes near the 37 mbQTLs discovered in our study (*q* < 0.05) to genes reported in four published microbiome GWAS data [19, 27, 54, 55]. The TwinsUK study, the largest human gut microbiome QTL study to date, reported 17 genes within 10 kb of 15 mbQTLs were associated with 15 different taxa (FDR < 5%). Blekhman et al. (2015) identified 34 exonic microbiome QTLs within 32 genes (*q* < 0.05) associated with taxa from within 12 Human Microbiome Project (HMP) body sites. Bonder et al. (2016) meta-analyzed three gut microbiome Dutch cohorts and identified nine loci associated with relative abundance and 33 with gene ontology terms and microbial pathways (*p* < 5 × 10^−8^). Lastly, Davenport et al. (2015) reported seven loci associated with six taxa (*q* < 0.05) in a gut microbiome GWAS in the Hutterites. Only one gene, Slit guidance ligand 3 (*SLIT3*) on chromosome 5, had multiple variants associated among these published studies (rs10055309 in TwinsUK and rs2163761 in Bonder et al. 2016). Overall, two genes with mbQTLs identified in this study at *q* < 0.05 have been previously reported in at least one other study: *SLIT3* [19, 55] and Sortilin related VPS10 domain containing receptor 2 (*SORCS2*) [55]. An additional seven genes identified using more relaxed thresholds (*q* < 0.10 in our study and all reported genes in other GWAS studies) are noted in Table 2 and Additional files 2, 3, and 4. Overall, there is little overlap between studies, likely reflecting the specificity of microbial communities to local environments and body sites, and the heterogeneity between studies with respect to sample size, geography, body sites, and the race, age and gender composition of the samples.

Nonetheless, three intronic variants in the gene *SLIT3* (LD *r* ^*2*^ < 0.05) on chromosome 5 were each associated with either the RA of an unclassified genus of family Clostridiaceae in TwinsUK (rs10055309) or the sitosterol degradation to androstenedione pathway (MetaCyc PWY-6948; pathway involved in plant-derived steroid degradation) in Bonder et al. (2015; rs2163761), and with the RA of genus *Dermacoccus* in the nasal vestibule in the summer in our study (rs77536542; *p* = 6.35 × 10^−8^; Fig. 4a). Both rs10055309 and rs2163761 were the most significant QTL reported in each study. *SLIT3* is a secreted protein that is widely expressed across many tissues with highest expression in skin, brain cerebellum, and lung [56]. *SLIT3* hypermethylation has been reported in a number of human cancers [56], and *SLIT3* expression is increased in LPS-stimulated macrophages in mice [57].

A variant 57 kb downstream of *SORCS2* on chromosome 4 (rs111354832) was associated in our sample with an unclassified genus of family Micrococcaceae (combined season nasal vestibule) and with *Kaistobacter* (nasal vestibule in the summer) while an intronic variant in *SORCS2* (rs10012347; LD *r* ^2^ = 0.17) was associated with the sitosterol degradation to androstenedione pathway (MetaCyc PWY-6948) in Bonder et al. (2016). Furthermore, an additional QTL in *SORCS3* (rs703462), a paralog of *SORCS2* on chromosome 10, was associated with *Peptoniphilus* in the combined nasopharynx sample (*q* < 0.10). SORCS genes are strongly expressed in the central nervous system, and their patterns of expression have been implicated in organ development in mice, including the development of the lung and nasal epithelial tissues [58]. The multiple associations of variants in *SLIT3* and *SORC* genes in the TwinsUK study, Bonder et al. (2016) and in our study suggest that these genes may play a role in modulating bacterial abundances in a genotype-specific manner across diverse body sites in humans.

## Discussion

Our study is the first to assess the role of genome-wide host genetic variation in shaping the human microbiome at the two upper airway sites. We first demonstrated reduced bacterial beta diversity between more closely related pairs of individuals and then discovered associated genetic variants at functionally related genes. These combined results indicate a significant role for host genotype in patterning microbial diversity in the nose.

Our results further suggest that the upper airway may be the site of important gene-environment interactions. In this context, host genotype at many loci may ultimately impact health and disease by modulating particular members of the microbial community. For example, a missense variant in fucosyltransferase 3 (*FUT3;* rs28362459) was strongly associated with decreased abundance of *Dermacoccus* (the nasal vestibule in the summer), a bacteria that is depleted in the skin of individuals with atopic dermatitis [35]. This SNP is predicted to be deleterious by both Polymorphism Phenotyping (PolyPhen) v2 [59] and Combined Annotation Dependent Depletion (CADD) [60] scores (0.997 and 15.12, respectively). Interestingly, the non-secretor phenotype, characterized by a null variant in another FUT gene, *FUT2,* and the resulting absence of ABH antigens in the mucosa in homozygotes for the null allele, influences the composition and diversity of the microbiome in the human intestinal tract [61, 62]. Moreover, variants in both *FUT2* and *FUT3* have been shown in GWAS to increase susceptibility to diseases associated with both mucosal surface pathobiology and microbiome composition, such as cystic fibrosis [39], Crohn’s disease [63], and ulcerative colitis [64]. Our study extends a role for fucosyltransferases to the nasal mucosal surface and further implicates host genetic influences on bacterial diversity at this site.

Four mbQTLs show effects on phylogenetically diverse phyla, and two were identified in different seasons and nasal sites. In particular, a missense variant in *PGLYRP4* (rs3006458) was associated with the abundance of genus *Aerococcus* (Firmicutes) in the nasopharynx in the summer and with family Micrococcacea (Actinobacteria) in the nasopharynx in the combined sample. The RAs of *Aerococcus* and Micrococcacea are only weakly correlated with each other indicating that these are likely independent associations. Moreover, the associations with phylogenetically distant bacteria and in different subsamples (summer vs. combined) suggest that *PGLYRP4* has pleiotropic effects over several organisms. Alternatively, the SNP identified in our study (rs3006458) could be tagging a haplotype with multiple variants that have independent effects on different bacterial abundances. The genomic region that includes the PGLYRP genes includes a cluster of genes implicated in epidermal barrier function [65], and SNPs in this region show extensive LD. However, independent evidence suggests that *PGLYRP4* may be the target gene of this association. The rs3006458-T allele, which is associated with lower RA of *Aerococcus* and Micrococcacea in our study, was associated with increased gene expression of the *PGLYRP4* gene in the epithelial and mucosal tissues (skin, small intestine, and esophageal mucosa) in the Genotype-Tissue Expression [66], and in the lung tissue in a separate eQTL study [67]. These tissues serve as physical barriers and provide innate immune functions essential for antimicrobial defense [68]. Collectively, these data suggest that host genotype at rs3006458 (or a variant in LD with rs3006458) regulates the expression in *PGLYRP4* in the skin, lung, and airway mucosa and functions to modulate bacterial abundance, possibly beyond the two genera identified in this study. The link between genetic variation in host PGRPs and microbiome abundance revealed in this study indicates that at least some of the important roles these proteins play in modulating communities of symbiotic organisms [69] are attributable to host genetic variation.

Eight of the mbQTLs identified in our study at a relaxed *q* < 0.10 influenced the abundance of more than one organism. Most were identified within the same season and nasal site, and four influenced the abundance of multiple closely related bacteria. For example, an intronic variant in *LRRC16A* (rs1543603) was associated with the abundance of five highly correlated genera of phylum Proteobacteria in the nasopharynx in the summer and co-occur in a larger network of 12 Proteobacteria and one Bacteroidetes. One possible explanation for this correlated network may be that these bacteria are physically interacting and their overall community structure is influenced by host genotype, but there may be other metabolic or physiological reasons why these bacteria co-occur. Although not much is known about *LRRC16A,* other proteins with leucine-rich repeat (LRR) domains, such as nucleotide-binding oligomerization domain receptors (NODs) and toll-like receptors (TLRs) [70], function as recognition receptors in innate immunity.

Although our study provides novel insights into host genetic influences on the nasal microbiome, there are some limitations. In particular, the size of our sample is relatively small for genetic mapping (77–88 individuals in seasonal analyses; 125 and 133 individuals in the combined). We reasoned that the reduced environmental heterogeneity among Hutterite individuals would enhance the effects of genetic variation and facilitate the detection of associated variants. While we were successful in identifying mbQTLs, we acknowledge that there are likely many more associations to be found in larger samples. A second limitation is the multiple testing burden that results from the high dimensionality of the microbiome. While we reduced the number of tests performed by mapping only genus level bacteria present in the majority of individuals, we only corrected for multiple testing within each study and did not correct for the 90 bacteria and six subgroups for which we performed mbQTL mapping. Although we used a fairly stringent threshold of genome-wide significance (*q* < 0.05), we acknowledge that some of our findings may be false positives. In addition, there is the possibility that samples from the nasopharynx may have been contaminated by the anterior nares; these regions are, indeed, anatomically and physiologically distinct. Our sampling method was motivated by the practicalities of field research and utilized standard clinical techniques. We note that only one study to date has surveyed the regional biogeography of the nasal microbiome; they included the anterior nares and two more posterior nasal sites (middle meatus and sphenoethmoidal recess, which are distinct from the nasopharynx, though also lined by respiratory epithelium) using a similar sampling technique [29]. Our findings of separation between the anterior and posterior nasal sites across some measures and variables (most prominently in the summer) are broadly consistent with their results, which show some differences and some overlap across sites. Nevertheless, precise spatial analyses of the nose require additional studies using alternative sampling techniques. Lastly, the genetic effects revealed by our study are context specific due to the many environmental and stochastic factors that affect microbiome composition and, therefore, challenging to replicate. For example, even within our study of a relatively homogenous population, we detected significant effects of season, age, and gender. In fact, most of the mbQTLs that we identified were specific to one season and demonstrate that even small temporal changes (6 months) in the RAs of bacteria within the same individuals can mask or enhance genetic effects. Although we did not formally replicate the results in independent populations, the identification of multiple intronic variants within the gene *SLIT3* in our study, the TwinsUK study [19], and Bonder et al. (2016) [55] bolsters confidence in the involvement of this gene in regulating microbial structure across multiple mucosal sites.

## Conclusions

Our study provides evidence for genetic contributions to modulating variability of the nasal microbiome, a trait that has been linked to a number of airway diseases [71]. Importantly, our findings support the concept that host genetic variation directly influences the expression or function of genes that are specifically involved in innate mucosal immunity pathways. Such a framework is consistent with previous reports showing that antimicrobial peptides [69, 72] and host immunity [73] are the key modulators of microbial defense in the mucosa. Our data further suggest that host genetic effects on immune genes modulate particular bacteria or the structure of whole microbial communities in the upper airways. We speculate that interactions between host genetics and microbiome structure or composition in the upper airway can influence dysbiotic tendencies that may predispose to respiratory disease and could be subject to intervention. Indeed, moving forward, more detailed analyses of the complex relationship between genetic variation in host mucosal immunity and the microbiome—captured here in a snapshot in the upper airway—are required to fully characterize determinants of an inherently dynamic microbial ecosystem. Such work could potentially identify targets for novel therapeutic strategies useful across a wide range of respiratory diseases.

## Methods

### Sample collection

Nasal brushings from Hutterites ages 16 to 78 from five colonies located in South Dakota, all within 14 miles of each other, were collected at two time points, winter (January/February 2011) and summer (July 2011), and from two nasal sites, the nasal vestibule and the nasopharynx. Samples from each of the two nasal sites were collected from opposite nares using sterile flock collection swabs (Puritan© 25-3316). The HMP anterior nares collection protocol [74] was used for the nasal vestibule, and the Center of Disease Control and Prevention (CDC) protocol for collection of nasopharyngeal swabs (http://www.cdc.gov/urdo/downloads/SpecCollectionGuidelines.pdf) was used to sample the nasopharynx. After excluding samples with low DNA yield, low sequencing read depth, antibiotic history within the prior 3 months, current use of steroid nasal spray, or missing genotypes, our final data consists of 133 individuals with nasal vestibule samples (87 summer and 80 winter) and 125 individuals with the nasopharynx samples (88 summer and 77 winter; Table 1).

### Sample DNA extraction library preparation and sequencing

Nasal brushes were immediately frozen at −20 **°**C following collection, shipped on dry ice, and stored at −80 °C. DNA extraction was carried out using the BiOstic® Bacteremia DNA Isolation Kit (12240–50). DNA concentration and purity were assessed using the Nanodrop 1000 spectrophotometer (Thermo Scientific, IL, USA). The 16S rRNA gene V4 region was amplified following conditions in Caporaso et al. protocol [75] using 62 different region-specific primers labeled with a unique 12-base Golay barcode sequence in the reverse primer. Final libraries were quality controlled prior to pooling with the Agilent Bioanalyzer DNA 1000 (Agilent Technologies, CA, USA). Libraries were pooled into 8 pools of 62 samples and sequenced on the HiSeq2000 platform (Illumina Inc., CA, USA) under a single end 102 base-pair protocol.

### Sequencing and taxonomic classification

Data was pre-processed using CASAVA 1.8.1. Following sample de-multiplexing, 617,909,462 sequence reads were processed using the Quantitative Insights into Microbial Ecology (QIIME) 1.8.0 toolkit [76]. Quality controlled reads were required to have an exact match to an expected barcode, zero ambiguous base calls, less than three consecutive low-quality base calls, and a minimum Phred quality score of 20 along the entire read. We used an open-reference OTU workflow where sequences were first clustered against the Greengenes May 2013 reference [77], and reads that did not cluster with known taxa (97% identity) were subjected to de novo clustering. Representative sequences were aligned using PyNAST version 1.2.2 [78], and the taxonomy of each OTU cluster was assigned with the *uclust* classifier version 1.2.22q. We applied an OTU abundance filter of 0.005% [79] to reduce spurious OTUs, removing 5.96% of total sequences and obtaining a final dataset of 563 OTUs.

### Data processing

#### Seasonal

For each of the four seasonal groups (the nasal vestibule in the summer and in the winter, and the nasopharynx in the summer and winter), a genus level RA table was calculated after subsampling reads to 250,000 per sample. Each bacteria’s RA was then quantile normalized using the *qqnorm* function in R. Next, PCA was performed using the *prcomp* function in R and each of the top 10 principal components (PCs, explaining ~76–78% of the variance) were tested in a linear model against technical covariates. In at least one of the seasonal groups, we identified correlations between one of the PCs with DNA concentration prior to PCR, final base pair fragment size and date of sampling (p < 0.001). PCR adapter barcode, library batch, order within library batch, and final library concentration were not significant. After regressing out the identified technical covariates from the normalized RAs, we performed PCA on the residuals and tested for associations between the top 10 PCs and biological covariates. Age and sex were significant and were therefore adjusted for in all subsequent analyses. Next, to reduce the burden of multiple testing in the seasonal mapping studies, we removed genera that were detected in fewer than 75% of individuals. This resulted in 78 genus level RAs in the nasal vestibule in the summer, 52 in the nasal vestibule in the winter, 90 in the nasopharynx in the summer and 59 in the nasopharynx in the winter.

#### Combined seasons

Although combining samples across seasons could introduce noise, it provides the largest possible sample size and consequently greatest power for genetic associations with bacteria that do not vary in abundance across seasons. Therefore, for each nasal site, we averaged the summer and winter genus level RA residuals obtained after quantile normalization and the regression of identified technical covariates for individuals with measurements during both seasons, or included the one season result for those with only one measurement (referred to as the combined sample). We selected all genus level bacteria present in at least 75% of individuals in either season, which resulted in 76 genus level RAs in the nasal vestibule and 90 in the nasopharynx. We performed PCA on the combined seasons matrix to verify variation among samples did not separate the combined samples from the samples with one seasonal measurement. Season of origin (summer, winter or averaged) was not correlated with any of the top 10 principle components in either nasal site (Additional file 1: Figure S5).

### Genotype data

The Hutterite individuals in our study are related to each other in a 13-generation pedigree that includes 3,671 individuals, all of whom originate from 64 founders. Using PRIMAL [26], an in-house pedigree-based imputation algorithm, whole genome sequences from 98 Hutterite individuals were phased and imputed to 1,317 Hutterites who were previously genotyped on Affymetrix arrays [80–82]. For mapping studies, we first selected 3,161,460 variants with genotype call rates greater than 95% in our sample and minor allele frequencies (MAF) > 0.10 in any of the 4 season/site subsamples. Next, we estimated LD in the Hutterite data using PLINK [83], and pruned variants for LD using an r^2^ threshold of 0.5, to yield a final set of 148,653 variants for mapping studies.

### Diversity metrics

Alpha diversity metrics at the species level (observed species, Shannon index and evenness) were calculated in QIIME [76] using the alpha_diversity.py script after subsampling reads from 1,000 to 10,000 every 1,000 reads, from 10,000 to 100,000 every 10,000 reads and from 150,000 to 250,000 every 50,000 reads. Each subsampling series was completed 10 times and rarefaction curves were plotted. Diversity metrics were averaged from the 250,000 read subsamples using the collate_alpha.py script and this metric was compared across seasons and nasal sites.

To calculate beta diversity, we first obtained the OTU table using *phyloseq* [84], quantile normalized OTU abundances using *qqnorm* in R and regressed out technical covariates (DNA concentration prior to PCR, final base pair fragment size and date of sampling). Next, we calculated pairwise Euclidean distance using the *vegdist* function in the R package *vegan*. We chose Euclidean distance as our beta diversity metric because this metric does not depend on raw relative abundance values for distance calculation. We wanted to correct our data for significant technical covariates, and Euclidean distance can be calculated on residuals after regressing out these technical covariates.

### Kinship associations to beta diversity

Pair-wise kinship coefficients were previously calculated by PRIMAL [26] using 271,486 variants genotyped on Affymetrix platforms. The average kinship coefficient between all pairs of individuals (*n* = 144) in our study was 4.51% (range 0.60–32.03%). We performed 10,000 permutations to assess the association between pairwise Euclidian distances and kinship coefficients in the combined season samples (nasal vestibule 8778 pairs, nasopharynx 7750). The *p* value is the number of times out of 10,000 permutations that the Spearman correlation of the permuted sequence pair was more extreme than the observed pair.

### Co-occurrence network analyses

We used SparCC [49] to calculate the nasopharynx in the summer correlation coefficients between all 90 genera tested in our mapping studies. We applied default settings and assigned *p* values calculated from 100 bootstraps. Co-occurrence networks were generated from the SparCC correlation matrix for genera with correlation *r* ^2^ > 0.75 and *p* < 0.01 (1/100 bootstraps). The network was generated using *igraph* R package, where nodes represent each genera and edges represent correlations between the genera above the applied threshold.

### Ingenuity Pathway Analysis of protein-protein interaction networks

We selected the closest gene to 1,413 variants (131 genes) with Hutterite linkage disequilibrium (LD) *r* ^2^ > 0.8 with the 108 mbQTLs (*q* < 0.10). To interrogate and visualize network associations, we used the Ingenuity Pathway Analysis Knowledge Base (IPA®, QIAGEN Redwood City, CA), limiting interactions to primary cells or tissues. The network scores generated by IPA are based on a right-tailed Fisher’s exact test comparing the observed and expected mbQTL genes present in a pathway relative to the IPA database.

## Additional files

Additional file 1: Supplemental materials (DOCX 1184 kb)
Additional file 2: Distributions of 166 genus level nasal microbiome relative abundances (XLSX 66 kb)
Additional file 3: QTL mapping results of nasal microbiome relative abundance (XLSX 216 kb)
Additional file 4: Nasal microbiome alpha diversity (XLSX 43 kb)
Additional file 5: QTL mapping results of nasal microbiome beta diversity (XLSX 50 kb)
Additional file 6: Nasal vestibule and nasopharynx SNP heritability estimates (XLSX 12 kb)

## Abbreviations

FDR: False discovery rate
FUT: Fucosyltransferase
HMP: Human Microbiome Project
LD: Linkage disequilibrium
LPS: Lipopolysaccharide
mbQTL: Microbiome quantitative trait loci
OTU: Operational taxonomic unit
PGLYRP: Peptidoglycan recognition protein
RA: Relative abundance
