# Supplementary material for: Host genetic variation in mucosal immunity pathways influences the upper airway microbiome
Source: Microbiome. 2017 Feb 1;5:16. doi: 10.1186/s40168-016-0227-5 (PMC5286564; doi:10.1186/s40168-016-0227-5)
Supplement: Additional file 1: — Supplemental materials (DOCX 1184 kb) [file 40168_2016_227_MOESM1_ESM.docx]

Host genetic variation in mucosal immunity pathways influences the upper airway microbiome

Catherine Igartua^1^, Emily R Davenport^1,2^, Yoav Gilad^1,3^, Dan Nicolae^1,3,4^, Jayant Pinto^5*^, Carole Ober^1*^

^1^Department of Human Genetics, University of Chicago, Chicago, IL

^2^Department of Molecular Biology & Genetics, Cornell University, Ithaca, NY

^3^Department of Medicine, University of Chicago, Chicago, IL

^4^Department of Statistics, University of Chicago, Chicago, IL

^5^Section of Otolaryngology-Head and Neck Surgery, Department of Surgery, University of Chicago, Chicago, IL

*These authors contributed equally

**Supplemental Materials**

**Supplemental Table S1: A. Distributions of 166 genus level nasal microbiome relative abundances.** RA distribution summaries of are presented for the nasal vestibule and the nasopharynx for the summer and winter sample. Min, minimum; Qu, quantile; Max, maximum. **B-C. Results of seasonal differences in genus level relative abundances.** 12 genera in the nasal vestibule and 15 in the nasopharynx differed in relative abundance (RA) by season, nine of which were different at both nasal sites. **D-E. Results of nasal site differences in genus level relative abundances.** No bacteria differed between nasal sites after multiple testing correction. P values are based on paired Wilcox rank sum tests calculated from individuals studied in both seasons or both sites. The percent of individuals with detectible bacteria (RA > 0) is presented in parenthesis. *Represents bacteria significant after correcting for 166 tests.

See Additional file 2 – Supplemental_Table_S1.xls

**Supplemental Figure 1: Nasal microbiome alpha diversity A. Rarefaction curves**. Sequencing reads were subsampled 10 times from 1,000 to 10,000 every 1,000 reads, from 10,000 to 100,000 thousand every 10,000 reads and from 150,000 to 250,000 every 50,000 reads (x-axis). **B. Alpha diversity by sex.** For both sexes, the number of species is lower in the nasal vestibule in the summer (light blue) compared to the nasopharynx in the summer (light green). Within females, alpha diversity is lower in the nasopharynx in the winter (dark green) compared to the nasopharynx in the summer (light green). Compared to males, females have lower number of species in the nasal vestibule in the winter (dark blue) and overall lower alpha diversity in the nasopharynx in the winter (dark green). P values correspond to a Wilcox rank sum test.

**Supplemental Figure S2: Correlation of alpha diversity with age in the nasopharynx in the summer.** Age is negatively correlated with Shannon index (p = 0.019) and evenness (p = 0.019), in the nasopharynx in the summer. Red line represents line of fit from a linear model.

**Supplemental Figure S3: Boxplots of beta diversity grouped by nasal site and between nasal sites.** For each of the nasal sites (panels 1 and 2), beta diversity (Euclidean distance) was lower in the summer (red) compared to the winter (blue) and between seasons for the same individual (purple) compared to all other pairs of individuals (green). In nasal site analyses (panel 3), beta diversity was lower for the same individual between nasal sites compared to all other pairs of individuals (summer: yellow vs. orange; winter: pink vs. brown). *Wilcox rank sum test p < 10^-7^.

**Supplemental Table S2: QTL mapping results of nasal microbiome relative abundance.** 108 microbiome quantitative trait loci (mbQTLs) were associated at a relaxed significance threshold of q < 0.10 with the relative abundance of 37 microbial taxa. Direction of effect is presented for the minor allele. Significant seasonal results are bolded. RA, relative abundance; Chr, chromosome.

See Additional file 3 – Supplemental_Table_S2.xls

**Supplemental Table S3: QTL mapping results of nasal microbiome alpha diversity.** One mbQTL was associated in the summer nasopharynx and six in the winter nasal vestibule (q < 0.10).

See Additional file 4 – Supplemental_Table_S3.xls

**Supplemental Table S4: QTL mapping results of nasal microbiome beta diversity.** The first 10 principal components of Euclidean distance (~44.42%-51.56% of the total variance) were mapped for the summer, winter and combined nasal vestibule and nasopharynx samples. 33 mbQTLs were associated at a q < 0.05 and an additional 98 mbQTLs at the more relaxed threshold of q < 0.10.

See Additional file 5 – Supplemental_Table_S4.xls

**Supplemental Table S5: Nasal vestibule and nasopharynx SNP heritability estimates.** Estimates for the combined season sample were calculated using GEMMA. PVE: percent variance explained; SE: standard error.

See Additional file 6 – Supplemental_Table_S5.xls

**Supplemental Figure S4: Locus and genotype plots for selected mbQTLs. A. rs3006458 is a mbQTL associated with lower RA of an unclassified genus of family Micrococcacea in the combined sample. B. rs1543603 is a mbQTL associated with higher RA of an unclassified genus of family Caulobacteraceae in the nasopharynx in the summer.** Variants on locus plot represent Hutterite variants with MAF > 10% prior to LD pruning. Numbers underneath each boxplot represent the number of individuals in each genotype class.

**Supplemental Figure S5: Principal component analysis (PCA) of genus level nasal vestibule and nasopharynx microbiome samples combined across seasons.** For individuals present in both seasons, residuals were averaged after quantile normalization and regression of technical covariates. In a linear model, PCs 1-10 did not correlate with season of origin (p >0.05).
